# Theory-based development of an implementation intervention to increase HPV vaccination in pediatric primary care practices

**DOI:** 10.1186/s13012-018-0729-6

**Published:** 2018-03-13

**Authors:** Jane M. Garbutt, Sherry Dodd, Emily Walling, Amanda A. Lee, Katharine Kulka, Rebecca Lobb

**Affiliations:** 1 Department of Medicine, Washington University, St. Louis, MO USA; 2 Department of Pediatrics, Washington University School of Medicine, Campus Box 8116, 660 S. Euclid Ave, St. Louis, MO 63110 USA; 3 Department of Pediatrics, University of Michigan, Ann Arbor, MI USA; 4 Department of Surgery, Washington University School of Medicine, St. Louis, MO USA

**Keywords:** HPV vaccine, Implementation strategies, CFIR, TDF

## Abstract

**Background:**

The national guideline for use of the vaccine targeting oncogenic strains of the human papillomavirus (HPV) is an evidence-based practice that is poorly implemented in primary care. Recommendations include completion of the vaccine series before the 13th birthday for girls and boys, giving the first dose at the 11- to 12-year-old check-up visit, concurrent with other recommended vaccines. Interventions to increase implementation of this guideline have had little impact, and opportunities to prevent cancer continue to be missed.

**Methods:**

We used a theory-informed approach to develop a pragmatic intervention for use in primary care settings to increase implementation of the HPV vaccine guideline recommendation. Using a concurrent mixed methods design in 10 primary care practices, we applied the Consolidated Framework for Implementation Research (CFIR) to systematically investigate and characterize factors strongly influencing vaccine use. We then used the Behavior Change Wheel (BCW) and the Theoretical Domains Framework (TDF) to analyze provider behavior and identify behaviors to target for change and behavioral change strategies to include in the intervention.

**Results:**

We identified facilitators and barriers to guideline use across the five CFIR domains: most distinguishing factors related to provider characteristics, their perception of the intervention, and their process to deliver the vaccine. Targeted behaviors were for the provider to recommend the HPV vaccine the same way and at the same time as the other adolescent vaccines, to answer parents’ questions with confidence, and to implement a vaccine delivery system. To this end, the intervention targeted improving provider’s capability (knowledge, communication skills) and motivation (action planning, belief about consequences, social influences) regarding implementing guideline recommendations, and increasing their opportunity to do so (vaccine delivery system). Behavior change strategies included providing information and communication skill training with graded tasks and modeling, feedback of coverage rates, goal setting, and social support. These strategies were combined in an implementation intervention to be delivered using practice facilitation, educational outreach visits, and cyclical small tests of change.

**Conclusions:**

Using CFIR, the BCW and the TDF facilitated the development of a pragmatic, multi-component implementation intervention to increase use of the HPV vaccine in the primary care setting.

## Background

There is a critical need for theory-based implementation interventions to improve the use of evidence-based practices in healthcare settings [1–4]. A conceptual framework that describes interactions between individuals, organizations, and the external environment can guide a comprehensive assessment of the implementation problem and identify important variables to consider when designing an implementation intervention. Improving use of evidence-based practices requires behavior change, and in order to design effective interventions and allow purposeful improvements when interventions fail, it is necessary to understand these behaviors in context [1–3, 5]. Models and theories of behavior and behavior change can be used to determine who and what needs to change and to identify behavior change strategies.

Until recently, this reasoned approach to development of implementation interventions was hindered by a bewildering array of implementation frameworks and psychological theories of behavior change. In the past 10 years, behavioral scientists have tried to simplify implementation frameworks and psychological theory for use by implementation researchers. Damschroder and colleagues developed the Consolidated Framework for Implementation Research (CFIR) to consolidate and unify key constructs from 19 published implementation theories [3]. The CFIR identifies five major domains of organizational context that influence successful implementation: characteristics of the implementation intervention, the inner setting (the context through which implementation will proceed), the outer setting (the context in which the organization resides), implementer’s characteristics, and the processes of implementation [3]. Michie and colleagues consolidated 33 theories of behavior and behavior change in order to develop a theoretical framework for understanding behavior change among health professionals [5–7]. Using consensus among experts, they identified a theoretical framework with 14 theoretical domains (or key theoretical constructs) covering the main factors influencing provider’s clinical behaviors and behavior change (Theoretical Domain Framework, TDF) [5, 6, 8]. These domains have been linked to a simplified model of behavior change, the COM-B model, using a “Behavior Change Wheel” (BCW). The COM-B model characterizes behavior change in terms of capability, opportunity, and motivation (COM-B). The BCW illustrates the interventions and behavior change functions that link the TDF to the COM-B model [2, 5, 7].

The Center for Disease Control and Prevention (CDC) national guideline for use of the human papillomavirus (HPV) vaccine is an evidence-based practice that is poorly implemented in pediatric practices [9, 10]. The HPV vaccine is an effective vaccine targeting oncogenic HPV strains. It is predicted to prevent over 90% of cancer attributed to HPV including cervical cancer and other genitourinary and oral cancers in men and women [11]. Importantly, cancer prevention requires vaccination prior to exposure to the sexually transmitted virus. To achieve this goal, the CDC guideline recommends completing the HPV vaccine series for girls and boys before their 13th birthday and giving the first dose at their 11- to 12-year-old check-up visit concurrent with other recommended vaccines [9, 12]. However, the vaccine is underused and opportunities to prevent HPV-related cancers are being missed [13]. In 2016, 10 years after the vaccine was first introduced in the U.S., only 49.5% of eligible females and 37.5% of eligible males aged 13–17 years had completed the vaccine series [11].

Theory-informed implementation interventions are urgently needed to increase use of HPV vaccine as recommended in the CDC guideline. Missing from the research is a comprehensive assessment of the implementation problem and theory-informed behavior change implementation strategies. Systematic reviews suggest that physician-level interventions such as audit and feedback and reminders and patient-level interventions such as education and reminder and recall have had at best a modest effect (~ 5% increase) [10, 14–16]. Provider-focused multi-component interventions were considered to be the most promising. Studies showing larger effects have been criticized for being methodologically lacking [10, 14–16]. Theory-informed, multi-component interventions may be more effective than prior interventions that failed to address all relevant barriers, and pragmatic approaches may provide scalable approaches to increase use of this important vaccine.

**Fig. 1:**
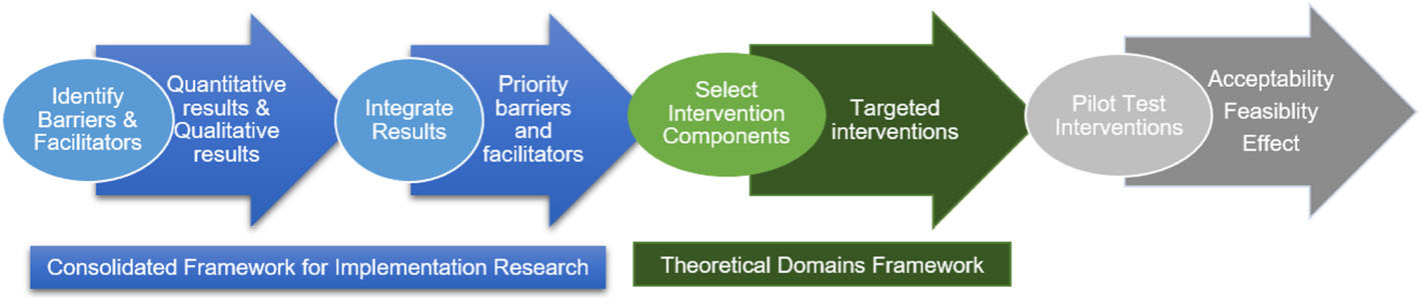
Steps for theory-based development of implementation intervention

This paper describes the systematic process of applying theory to develop a pragmatic intervention to increase use of HPV vaccine as recommended in the CDC guidelines. We used a multi-step approach (Fig. 1). First, we applied the CFIR [3] to systematically identify barriers and facilitators to completing the vaccine series by age 13 and identify priority barriers and facilitators in a broad sample of pediatric primary care practices. Then, we used the BCW and the TDF [6, 7] to identify targeted behaviors and select interventions with the strongest potential to increase adherence to vaccine guidelines while reducing barriers to implementation. This systematic attempt to develop an intervention to implement clinical guidelines for patient care was within the context of the healthcare team and practice setting [1]. We selected intervention components and the modes for delivery by considering what was feasible and acceptable to primary care providers working in independent offices. These components were combined to form a cohesive intervention for future evaluation.

## Methods

The study was approved by the Washington University Human Research Protection Office and was guided by an Advisory Board of three pediatricians, one pediatric nurse practitioner, and two parents. The Advisory Board assisted in the interpretation of study findings and the development of the intervention.

### Study participants

We invited all members of the Washington University Pediatric and Adolescent Ambulatory Research Consortium (WU PAARC) to participate. WU PAARC is a practice-based research network of 79 community pediatricians and 6 pediatric nurse practitioners associated with Washington University. The 16 providers from ten practices who volunteered to participate comprised the study sample. Each participant provided written consent and received $50 for completion of the semi-structured interview as a token of appreciation for their time.

### Identification of barriers and facilitators for HPV vaccine use

Guided by the CFIR, we used qualitative methods to identify barriers and facilitators to guideline-recommended HPV vaccine use and quantitative methods to identify vaccine coverage rates. We then integrated results from the qualitative and quantitative methods to identify theoretical factors that distinguished HPV coverage [17].

#### Collection and analysis of qualitative data

The provider interview guide addressed the main CFIR constructs and included both general and specific open-ended questions. The interviews were designed to last about 30 min and were conducted at the practices between January 27, 2016, and May 24, 2016, by the principal investigator (JG) and co-investigator (EW). All interviews were digitally recorded and transcribed verbatim by trained transcriptionists. The transcripts were organized using NVivo software, and each transcript was analyzed using consensual qualitative research methods with multiple analysts from diverse disciplines [18].

#### Collection and analysis of vaccination data

For each provider, we abstracted gender, date of birth, date of vaccine, and type of vaccine (HPV, tetanus, diphtheria, and pertussis, Tdap, and meningococcal, MCV4) from the chart of eligible adolescents. These included 11- to 15-year-old girls and boys who attended at least one office visit from January 1, 2014, to December 31, 2014. For each practice and provider, we computed the overall coverage with HPV vaccine as the percentage of girls and boys who completed the vaccine series (requiring three doses in 2014) by their 13th birthday (a HEDIS measure for adolescent care).

#### Integrated analysis of qualitative and vaccine coverage data

For each transcript, ratings were assigned that reflected the valence (positive or negative influence) of each CFIR construct on implementation of HPV vaccine according to the CDC guideline. This was done using a consensus process. We examined rating patterns within and across providers in the lowest and highest tertile groups for HPV vaccine coverage to identify barriers and facilitators and constructs that distinguished between groups [18]. Using maximum variation sampling based on coverage (i.e., quantitative findings) enabled us to highlight qualitative results that differed by coverage [18].

### Development of the implementation intervention

We used the TDF and the BCW to guide selection of the implementation interventions. The TDF is what Sales et al. describes as a fully developed theory because it answers the following questions: Why do people behave as they do? Given the way they behave, what would motivate them to change behavior [1]? The TDF posits that behavior change involves three essential conditions: capability, opportunity, and motivation. We organized findings from the integrated analysis by these three essential conditions and then matched the distinguishing factors to the behavioral antecedents, and interventions mapped to the respective condition in the TDF. We used the work by Michie et al. and by Powell et al. to help us to derive the intervention strategies [19–21]. Finally, we combined intervention components into a cohesive intervention for future testing.

## Results

### Identification of barriers and facilitators for HPV vaccine use

#### Study participants

Sixteen providers (15 pediatricians and one pediatric nurse practitioner) participated in both the qualitative and quantitative studies. Five were male, 11 were female, 13 were Caucasian, 2 were African American, and one was Asian. The 16 providers were drawn from ten practices, nine of which had multiple providers (2–6 providers per practice). Eight practices used an electronic medical record (EMR).

#### Interviews

A brief overview of the qualitative data analysis within the CFIR framework is provided here.

All providers were aware of the CDC recommendations to complete the HPV vaccine series by age 13 and universally assumed responsibility for vaccine delivery. Providers typically recommended initiation and completion of the HPV vaccine series during 11- to 12-year-old well visits; however, the strength of this recommendation varied. Most providers felt that information about vaccine benefits was needed to counter the misinformation parents received from the media and other sources. To meet this perceived need, providers routinely informed parents that HPV vaccine prevents cancer and sometimes they mentioned the prevention of warts. Providers varied in sharing personal information about vaccinating their children or grandchildren. Some provided the information routinely while others only provided this information if asked. All providers used the vaccine information sheets (VIS) from the CDC to provide parental education. Whenever parents wanted to know which of the available vaccines at 11- to 12-year-old well visits were mandatory for school attendance, providers routinely recommended the HPV, Tdap, and MCV4 vaccines, but suggested HPV vaccine was optional.

Delivery of HPV vaccine fit well with existing workflows in practices that delivered Tdap and MCV4 to all adolescents at the 11- to 12-year-old check-up visits, but required an additional visit when vaccination was delayed. Most providers were unaware of how their medical assistant or nurse approached scheduling follow-up doses with parents, and teamwork to deliver the vaccine series was uncommon. All providers offered vaccine-only visits and follow-up doses at acute care visits, and few practices routinely booked follow-up appointments or made reminder calls to parents. Most often the responsibility to schedule the second and third doses was left to the parent.

Commonly reported difficulties with providing HPV vaccine included taking longer to discuss HPV vaccine than other vaccines and dealing with the resistance and hesitancy of parents. Many felt that they could not persuade hesitant parents to provide HPV vaccine for their child and some preferred to delay discussion of prevention of a sexually transmitted cancer until the child was older and the parent was more accepting. Despite commonly experiencing challenges with giving HPV vaccine, few providers reported efforts to improve the system of vaccine delivery and none monitored vaccine coverage.

#### Vaccine coverage

In 2014, across all study sites, 13.9% of 4592 eligible teens had completed the HPV vaccine series before their 13th birthday (16.9% girls, 11.3% boys). This metric varied among practices from 2.3% to 25.5%. In the five practices where more than one provider participated in the study, this metric varied four- to fivefold among providers within the same practice. Due to this intra-practice variation among providers, we chose to analyze the integrated data at the level of the individual provider rather than at the level of the practice.

#### Integrated analysis

We identified fourteen constructs that distinguished between providers with higher versus lower HPV vaccine coverage that span the five domains of the CFIR. They are summarized and characterized as facilitators and barriers to guideline-recommended vaccine use in Table 1.

**Table 1:** Summary of distinguishing factors for completion of HPV vaccine series by 13th birthday characterized using the CFIR(1)

CFIR domain	Construct	Effect
Intervention characteristics	Relative advantage—at age 11–12 (increased immunogenicity, completion of series before risk, access to at-risk population)	Facilitator
	Adaptability—use older age of initiation	Barrier
Outer setting	Patient needs and resources—optional as not mandated by school	Barrier
	Peer pressure	Barrier
	External policy and incentives—financial incentives for series completion, e.g., meaningful use	Facilitator
Inner setting	Networks and communication—communication to coordinate implementation of 3 doses across staff and providers	Facilitator
	Readiness for implementation—leadership engagement in system level improvements and use of available resources, e.g., EMR alerts, outreach calls	Facilitator
Characteristics of individuals	Knowledge and beliefs—perceive value to completing by age 13	Facilitator
	Knowledge and beliefs—perceive value to bundling	Facilitator
	Self-efficacy—confident to strongly recommend vaccine and to convince hesitant parents	Facilitator
	Self-efficacy—enthusiastic about HPV vaccine	Facilitator
	Readiness to change—has made personal efforts for improvement	Facilitator
Process	Planning—discuss/implement changes for increased vaccine use	Facilitator
	Engaging—involve staff in meaningful problem-solving	Facilitator
	Executing—strong recommendation, routinely provide at age 11/12, bundle 3 vaccines	Facilitator
	Reflecting and evaluating—with a view to making changes	Facilitator

*CFIR* Consolidated Framework for Implementation Research

### Development of the implementation intervention

From the CFIR analyses, we found that most distinguishing factors between higher and lower coverage related to provider characteristics—their lack of buy-in to vaccination by age 13, their lack of confidence to address parental hesitancy, and poor communication skills to promote timely HPV vaccine use. Also, coverage was higher in practices with co-ordination between the provider and staff to ensure opportunities for vaccination were not missed. We concluded that the provider was the one whose behavior needed to change to increase vaccine coverage, and identified three targeted behaviors: (1) recommend getting the HPV vaccine in the same way and at the same time as other adolescent vaccines; (2) answer parent’s questions about the vaccine with confidence; and (3) work with staff and providers to develop a vaccine delivery system using all possible resources.

As illustrated in Table 2, behavior change strategies were chosen to influence the determinants of the three essential conditions of behavior changes from the BCW (i.e., capability, opportunity, and motivation), address the distinguishing barriers to implementation, and incorporate the distinguishing facilitators from Table 1. To increase capability, the intervention is designed to improve provider’s knowledge, communication skills, and self-efficacy regarding implementing guideline recommendations. Audit and feedback of vaccine coverage data will be used to increase motivation, and opportunity to increase vaccine use will occur through development of an integrated system for vaccine delivery within the practice. Strategies to change physician behavior will include providing information, communication skill training with graded tasks and modeling, action planning, goal setting, and social support. These strategies have been combined in an implementation intervention to be delivered using educational outreach visits, use of cyclical small tests of change, and practice facilitation (Table 2). These approaches were chosen as they have been shown to be effective in primary care practices that lack resources needed to implement change processes [22–24].

**Table 2:** Selection of behavior change techniques and implementation change strategies to increase HPV vaccine use

COM-B factor from the BCW [2]	Potentially modifiable determinant of behavior, i.e., barrier or facilitator from CFIR-guided analysis [3]	Theoretical domain and techniques for behavior change from TDF-guided analysis [20]	Implementation strategy [19]
Capability	Unaware of all benefits to the teen and practice of vaccination at 11/12-year check-up vs. deferring vaccine until older (intervention characteristics, outer setting)	Knowledge • Provide information about benefits of HPV vaccine at 11/12-check-up for teen and for practice	Develop and distribute educational materials for provider Conduct educational outreach visits
Capability	Lack of ability to effectively recommend HPV vaccine for use at the 11/12-year check-up (characteristics of individuals, outer setting, and process)	Skills • Model/demonstrate communication strategy • Rehearse use of communication strategy • Set goals for use of communication strategy and monitor behavior • Undertake graded tasks	Develop and distribute educational materials For providers: 4-part communication strategy • Make a strong recommendation • Provide simple responses for common questions • Provide personal information about vaccine use if appropriate • Implement a follow-up plan if parent is hesitant For parents: brochures/posters Test components of communication strategy in cyclical small tests of change using practice facilitation
Motivation	No or limited interest in changing approach to HPV vaccine delivery (characteristics of individuals, inner setting)	Action planning • Set goal for change • Planning the change	Audit and provide feedback of HPV vaccine coverage Promote adaptability for target age to encourage participation Practice facilitation
Motivation	Low self-confidence to provide strong recommendation, address parental concerns and deal with hesitant parents to allow timely use of vaccine (characteristics of individuals)	Belief about consequences • Undertake graded tasks. Use problem-solving, decision-making and goal setting • Process for encouragement and support • Provide feedback of improvement Social influences • Process for encouragement and support	Test components of communication strategy in cyclical small tests of change using practice facilitation Tailor implementation strategies Practice facilitation
Opportunity	Lack of vaccine delivery system (inner setting, process)	Environmental context and resources • Environmental changes to process of HPV vaccine delivery using all available resources	Conduct educational outreach visits Reminders Practice facilitation Conduct cyclical small tests of change

*BCW* Behavior Change Wheel, *CFIR* Consolidated Framework for Implementation Research, *TDF* Theoretical Domain Framework

#### Tools

Educational materials for the provider include a communication strategy to help providers present information in a more deliberate and impactful way to promote HPV vaccination before the preteen becomes sexually active.

Educational materials for the parent were developed as those currently available to provide information about HPV vaccine were judged to be inadequate by the Advisory Board. Specifically, our parent advisors felt the CDC brochures contained too much information and would not be read and providers wanted a generic brochure that could be used for girls and boys. Guided by the Advisory Board, we worked with the Health Communication Research Laboratory at the George Warren School of Social Work at Washington University to develop posters and a brochure to provide important information about HPV and HPV vaccine and to promote vaccine use. We tested the acceptability of these materials with a sample of 21 parents recruited from study practices and revised them to include their suggestions for improvement and address their concerns.

## Discussion

National recommendations for new vaccinations are not usually packaged for easy implementation and primary care providers continue to face considerable barriers when trying to implement the adolescent immunization schedule for HPV vaccine [25–28]. By using the CFIR to systematically examine the complex web of factors that influence the use of this important vaccine in the primary care setting, we identified facilitators and barriers to following national guidelines across all five domains of the conceptual framework. Accepting vaccination by age 13 was a critical aspect of providers’ ability and confidence to recommend the vaccine, address parental hesitancy, and promote timely HPV vaccine use. Other opportunities for improvement included better teamwork to share the work of vaccine delivery and increase efficiency and ensure opportunities for vaccination were not missed.

Our findings informed a theory-based, multi-component implementation strategy to change provider behavior regarding vaccine use. Using the TDF, we identified behavioral change targets and behavioral change approaches for providers. The intervention includes audit and feedback of vaccine coverage data to motivate providers to change their approach to deliver HPV vaccine. Several features of the audit and feedback component are considered “best practices” [29]. These include using recent, individual-level data, providing feedback in several different formats (table, graph, oral), and repeating feedback cycles. At a brief educational outreach visit, information about the benefits of using the 11- to 12-year-old check-up visit to initiate the vaccine series, both for the patient and for the practice will be shared. The theory-based communication plan provides key elements to guide practitioners’ conversations about HPV vaccine and facilitate providing a strong and persistent recommendation to complete the vaccine series by age 13. As most independent primary care practices lack the infrastructure and support for practice change, practice facilitation will be used to assist providers to implement the change process and to engage staff in the process [22]. Our hope is that this multi-component intervention designed to address multiple barriers will have a greater and more sustainable effect on HPV vaccine use than prior interventions focused on one or two barriers.

Several features of the proposed intervention are innovative and may inform implementing behavior change in primary care practice. We developed a simple, theory-based communication strategy that builds on established behaviors used to recommend other childhood vaccines. The implementation approach will allow providers to practice their communication about HPV vaccine, an effective strategy for learning new skills [30]. Few studies have tried to improve the quality of provider communication. Training providers to use an announcement approach has been shown to increase HPV vaccine coverage by 5% [31], but the broader literature of message framing has yielded mixed results [32]. Also, the intervention will allow autonomy for providers to select the target age for vaccine initiation with the goal to complete the series by age 13 to 15. Autonomy is important for intrinsic motivation and may be important to engage providers in the change process [33]. Involving other staff members in the system of care may be a pragmatic, alternative approach to EMR-reminders [28] and other strategies that may not be scalable without support. Practice facilitation has been used successfully to implement evidence-based interventions in primary care practice that lack the resources to engage in the change process [22].

Our study is limited in several respects. Our sample consists of a small number of providers from one geographic area and may not be representative of other populations. However, we believe the study sample is representative of providers working in small-scale, independently run community-based pediatric practices in our community with considerable variation in the context of how the providers practiced. In this setting, we were unable to examine the effect of delivery systems or practice leader engagement on vaccine use. Completion of the three-dose series before the 13th birthday was low among participants but is consistent with concurrent national data for this age group [34]. The time span for collecting the quantitative and qualitative data were different (2014 and 2016 respectively). National data show the increase in HPV vaccine coverage from 2014 to 2016 was minimal (about 5%) [11, 34, 35], but if changes in coverage were not equal across providers, then our assessment of barriers and facilitators may be inaccurate.

## Conclusions

In conclusion, study and national data suggest that widespread efforts to increase HPV vaccine use are urgently needed. We used the CFIR, the TDF, and BCW to systematically examine the use of this vaccine in independent primary care practices, identify targeted provider behaviors for change, and guide our selection of behavior change components needed to increase HPV vaccine use according to guideline recommendations. This theory-informed, multi-component implementation strategy merits further investigation. After pilot testing to assess feasibility, we are planning to seek funding to complete a rigorous evaluation in a clinical trial in the primary care setting to evaluate the effectiveness of this implementation strategy to make large improvements in HPV vaccine use.

## Abbreviations

CDC: Centers for Disease Control and Prevention
EMR: Electronic medical record
HEDIS: Healthcare Effectiveness Data and Information Set
HPV: Human papillomavirus
MCV4: Meningococcal B vaccine
NCATS: National Center for Advancing Translational Sciences
NIH: National Institutes of Health
Tdap: Tetanus, diphtheria, and pertussis vaccine
U.S.: United States
VIS: Vaccine information sheet
WU PAARC: Washington University Pediatric and Ambulatory Research Consortium
